# Autonomic factors do not underlie the elevated self-disgust levels in Parkinson’s disease

**DOI:** 10.1371/journal.pone.0256144

**Published:** 2021-09-02

**Authors:** Vasileia Aristotelidou, Marianna Tsatali, Paul G. Overton, Ana B. Vivas

**Affiliations:** 1 Department of Psychology, University of Sheffield, Sheffield, United Kingdom; 2 Greek Alzheimer Association Day Care Centre “Saint John”, Thessaloniki, Greece; 3 Department of Psychology, CITY College, University of York Europe Campus, Thessaloniki, Greece; National Institutes of Health, UNITED STATES

## Abstract

**Introduction:**

Parkinson’s disease (PD) is manifested along with non-motor symptoms such as impairments in basic emotion regulation, recognition and expression. Yet, self-conscious emotion (SCEs) such as self-disgust, guilt and shame are under-investigated. Our previous research indicated that Parkinson patients have elevated levels of self-reported and induced self-disgust. However, the cause of that elevation–whether lower level biophysiological factors, or higher level cognitive factors, is unknown.

**Methods:**

To explore the former, we analysed Skin Conductance Response (SCR, measuring sympathetic activity) amplitude and high frequency Heart Rate Variability (HRV, measuring parasympathetic activity) across two emotion induction paradigms, one involving narrations of personal experiences of self-disgust, shame and guilt, and one targeting self-disgust selectively via images of the self. Both paradigms had a neutral condition.

**Results:**

Photo paradigm elicited significant changes in physiological responses in patients relative to controls—higher percentages of HRV in the high frequency range but lower SCR amplitudes, with patients to present lower responses compared to controls. In the narration paradigm, only guilt condition elicited significant SCR differences between groups.

**Conclusions:**

Consequently, lower level biophysiological factors are unlikely to cause elevated self-disgust levels in Parkinson’s disease, which by implication suggests that higher level cognitive factors may be responsible.

## Introduction

Parkinson’s disease is a progressive neurodegenerative disorder of unknown aetiology, with a prevalence of .1-.2% in the general population, rising to 1% among people above 60 year of age [1]. The disorder affects all domains, motor, cognitive and emotional [2]. Parkinson’s disease is typically characterised by the motor symptoms of resting tremor, “cog-wheel” rigidity and bradykinesia [3–6]. Affective and cognitive symptoms, such as depression, anxiety and dementia have received far less attention and are often underestimated as peripheral although they are associated with high societal and healthcare costs [7].

Amongst the cognitive symptoms, deficits in recognition and expression of basic emotions (e.g., fear, disgust, sadness, happiness etc.) have been relatively well researched and documented [5, 6, 8–11]. For instance, there is high comorbidity between Parkinson’s disease and alexithymia [12–15], and studies have reported reduced facial and verbal expressivity [16–21], difficulties in emotional regulation [22–24] and emotion recognition [25] in patients with Parkinson’s disease. However, we know very little about how more cognitively complex emotions, namely self-conscious emotions (SCEs), are affected in these patients.

SCEs are different from basic emotions in that they require self-evaluation/appraisal in relation to other’s feedback [26–29]. These emotions (e.g., shame, guilt, self-disgust) are thought to play a key role in social regulation and adjustment by suppressing and promoting socially undesirable and desirable behaviours, respectively [30–34]. Research has suggested that this category of emotions, unlike basic emotions, involve sophisticated cognitive processes such as self-awareness [35–37] and the ability to recognize one’s own and also others’ mental states [38]. In this study, we assess the emotions of shame [26, 39] and guilt [40], which are relatively well studied, and self-disgust which has recently attracted scientific attention [41]

Shame refers to a negative evaluation of the whole self as “inadequate”, in other words, the person embodies the feeling of a “flawed self” [26, 39]. Subsequent behaviour involves hiding and isolating the self [26, 39]. During shame, empathy towards the self is supplanted by a feeling of self- distress, leaving the self exposed [40]. Abnormally heightened levels of shame have been associated with poorer anger and aggression management skills [42], depression [43], substance abuse [44], personality disorders [45], post-traumatic stress disorder [46], anxiety disorders [47], eating disorders [48] and suicidal/self-injury idealization [49].

On the other hand, guilt refers to a part of the self visible and vulnerable to judgment from the society [50]. In effect, one’s behaviour is considered to be inappropriate. The ultimate goal of guilt is to make the self a better, more productive and valued member of society, so it is powerfully associated with the feeling of empathy towards others. Higher trait levels of guilt have been associated with altered body language and affective social interactions [51]. When blended with other self-blaming emotions [52], excessive traits of guilt can facilitate affective disorders. Depression was once characterized as the “constant feeling of inappropriate guilt” [53]. On the other hand, the absence of remorse has been shown to independently predict aggressive conduct and antisocial disorders [54, 55].

Finally, a third negative SCE, self-disgust, refers to feelings of repulsiveness and loathing directed at the self [39, 56, 57]. From early on, self-disgust was proposed to act as a mediator in several psychiatric disorders [27]. Elevated levels of this emotion appear to mediate the relationship between dysfunctional cognition and depression [58], and are associated with social anxiety [59], psychoticism [60], eating disorders [61], obsessive compulsive disorder [62], and decreased levels of psychological wellbeing [63].

In relation to Parkinson’s disease, research suggests that self-awareness, a key cognitive process in SCEs, is altered [64–68]. Specifically, reduced self-awareness of memory (dys)function was positively correlated with disease severity, degree of memory decline and cognitive control deficits [69]. These results are in line with reports of anosognosia in Parkinson patients, who are unable to recognize and precisely express their physical symptoms [65]. Vann-Ward and colleagues [68] suggest that the concept of self is potentially maladaptive in Parkinson’s disease. Specifically, newly diagnosed patients are unable to adjust and preserve self-strategies such as the ability to develop relationships, envision the future, cope with everyday emotional discrepancies, perform self-evaluation tasks and have goals and aspirations.

Thus, it is not surprising that the limited research so far has reported altered SCEs in Parkinson’s disease. For instance, it has been suggested that Parkinson’s disease can be characterized as a “problem of shame”, and that increased shame may be associated with altered dopamine activity in key brain areas (e.g., prefrontal cortex and anterior cingulate cortex) underlying cognitive and affective processing [70]. Furthermore, guilt, as part of the psychotic symptomatology in Parkinson’s disease [71, 72], often appears as a subtype of hallucinations/delusions of the type “I am a sinister, I am guilty”. Other studies report feelings of guilt due to the disease’s progression and due to the extended disability of the patient [73, 74]. Self-disgust on the other hand, until very recently, was completely overlooked in Parkinson’s disease. In a recent study by Tsatali and colleagues [75], patients with Parkinson’s disease were found to have increased levels of self-reported and experimentally induced self-disgust, as compared to matched healthy controls, and when controlling for the confound effects of depression and anxiety. In contrast, self-reported and experimentally induced levels of shame and guilt were similar to those of the control participants. In the present study, we analysed physiological responses recorded during the experimental induction of SCEs, from the same group of patients and matched controls, to further investigate why self-disgust is elevated in Parkinson’s disease (see Tables 2 & 3 in [75] for differences between patients and controls in self-reported and experiementally induced levels of SCEs).

Schachter and Singer [76] were the first to introduce the two-factor theory of emotion, suggesting that the experience of basic emotions can be decoded as the interaction of physiological arousal and cognitive appraisal. Later on, Ekman and colleagues proposed that emotions could be related to distinct autonomic responses of the sympathetic and parasympathetic nervous system [77, 78]. Nowadays, this hypothesis is widely accepted [79–82]. Specifically, heart rate variability (HRV), or in other words, time and speed fluctuations between heart beats, refers to a metrics system of neurocardiac homeostasis [83], and HRV in the higher frequency band is considered to be an index of parasympathetic activity [84, 85]. In terms of basic emotions, elevated HRV seems to be elicited by happiness, anxiety, anger, contamination disgust, and intense sadness [86], while HRV decreases with disgust and mild sadness [87, 88]. Whilst high frequency band HRV is considered to be an index of parasympathetic activity, galvanic skin response (alterations in the electrical properties of the skin) depends almost solely on sympathetic activation [89]. The majority of studies report elevated levels of skin conductance response -SCR- to basic emotions, regardless of valence (e.g., fear, anger, disgust, happiness) [90]. In addition, enhanced skin conductance is also linked with successful emotional engagement, feelings of rejection, emotional distress and anticipation [91].

The majority of studies investigating physiological responses to emotional experiences have focused on basic emotions, and thus our knowledge on physiology of SCEs is scarce. As expected, the experience of SCEs also activates the autonomic nervous system, but research suggests a more general physiological arousal, which lacks emotion type-specificity [37, 92, 93]. Van ‘t Wout et al. [94] used the Ultimatum Game paradigm (unfair offers condition) to induce guilt in healthy participants while skin conductance was measured. The study reported significantly higher skin conductance response to rejections of unfair offers than to acceptance of fair offers. The authors concluded that participants experienced more emotional arousal when confronted with an unfair offer, but increased SCR could have resulted from the attempt to down-regulate guilt. Fourie et al. [95] found decreased HRV and increased SCR, during narration-induction of guilt relative to a neutral condition, in healthy participants. Accordingly, Pennebaker and Chew [96] reported that behavioural inhibition (mostly facial expression and voice tonality) to achieve deception, during a guilt induction paradigm, was associated with phasic increases in skin conductance. To our knowledge, there are only two studies investigating the physiology of shame. Kassam and Mendes [97] found higher HRV during a shame induction paradigm involving a mathematical task, compared to a neutral condition. Likewise, Harley et al., [98] reported a positive correlation between skin conductance level and shame, after completing a diagnostic reasoning task. Only one study has measured HRV in a self-disgust inducing mirror paradigm in participants with Body Dysmorphic Disorder and controls [99]. Although there were no differences between the groups in HRV and self-reported self-disgust, HRV was increased in 2 (out of 5) of the mirror inducing trials in the control participants. Thus, although the evidence so far is very limited, induction of guilt and shame seems to be associated with an increase in skin conductance, whilst HRV may increase (shame, self-disgust) or decrease (guilt).

In this study we tested the hypothesis that increased levels of experimentally induced self-disgust (narrations and self-photo) in Parkinson’s disease patients, relative to matched healthy controls [75], may result from altered physiological responses in patients. To do so, we analysed HRV (high frequency band) and SCR (amplitude) data that were obtained from the same group of patients and their matched controls, during the emotion-induction paradigms. We analysed both measures, and in addition created a composite score of the overall physiological response [100, 101]. If increased levels of self-disgust in Parkinson patients result from a heightened physiological response to this specific emotion, then we should find heightened physiological scores for the self-disgust induction conditions in the patients as compared to the matched controls. Furthermore, since the patients and control participants did not significantly differ in shame and guilt levels, there should not be significant differences in physiological responses between the groups for the shame and guilt induction conditions.

## Materials and methods

### Participants

Physiological data were analysed from the 40 patients with Parkinson’s disease (17 males and 23 females, with average age 71.73, SD = 9.93) and 40 controls (18 males and 22 females, with average age 71.87, SD = 9.02; matched for age and educational level) included in Tsatali et al. (75; see Table 1 for demographic and clinical characteristics of the patients and the control participants). The inclusion criteria for the control participants were: (i) a score in the Mini-Mental State Examination [102] (MMSE) equivalent to or higher than 24, (ii) absence of psychiatric disease or sustained head trauma, as self-reported by the participants, (iii) absence of alcohol, or drug or any other substance addictive behaviour, as self-reported by the participants, (iv) no history of hypothyroidism, which can affect skin conductance [103]. In the procedure, all the participants were instructed to breathe freely.

The inclusion criteria for the patients were: i) a Parkinson’s disease diagnosis according to the UK Parkinson’s Disease Society Brain Bank Clinical Diagnostic Criteria [104], ii) mild or moderate stage of disease progression, based on the UPDRS, iii) a MMSE examination with an outcome equal to or more than 24, iv) absence of any underlying mental or psychiatric disorder, or sustained head trauma; and v) absence of alcohol, or drug or any other substance addictive behaviour. Prior to the experimental procedure, patients with Parkinson’s disease were clinically evaluated by a neurologist. Almost all patients were under medication (combinations of levodopa, inhibitors of dopamine catabolism and dopamine receptor agonists) at the time of the study. Since the Parkinson patients had significantly higher anxiety and depressive symptoms than control participants (see Table 1 in 75), we included the total score of the *Hospital Anxiety and Depression Scale* as a co-variate in the analyses. ANCOVA analyses were reported only when there was a change in the results.

From the initial sample of eighty participants, SC and HRV were reliably extracted from 49 (24 PD, 25 HC) and 58 (24 PD, 34 HC) in the narration and photo paradigms, respectively. The remaining participants were excluded because the signal was not of sufficient quality due to noise that could not be eliminated [119].

The study was approved by the University of Sheffield Ethics Committee, and all participants provided their written consent.

### Measures and procedure

#### Narration emotion induction paradigm

Participants were asked to narrate orally past personal experiences in which they had felt guilty (Guilt narration), ashamed (Shame narration) and self-disgusted (Self-disgust narration). In the control condition (neutral narration), participants were asked to narrate what they did the day before, describing only the facts (see 75 for the detailed instructions). No time limit was set.

As the narrations were completed, participants were asked to report how they felt by using a Visual Analogue Scale (VAS) from 0 (Not at all) to 100 (Extremely) for the target emotion (self- disgust, guilt and shame) and other non-target emotions (anger, happiness and sadness), as well as their arousal levels.

#### Photo induction paradigm

As described in Tsatali et al., [75], two consecutive images were presented to participants, one of themselves (sitting in a chair in a neutral pose) and a neutral one acquired from the IAPS (International Affective Picture System, 2005). The participants were told to look carefully at the images. Each image was presented for 3 s, and followed by a blank screen for 20 s. Then, the participant was asked to report how they felt using a VAS, as described above. The order of the images (neutral and self) was counterbalanced across participants.

### Physiological data analysis methods

Before the beginning of the emotion induction paradigms, participants sat in a chair and the electrocardiogram (ECG) and electrodermal activity (EDA) sensors were applied. A Nexus wireless portable physiology recording device (Mind Media Nl, 2008 V2) was used to record the physiological data. The Nexus device was connected to a computer via Bluetooth, and heart physiology and skin conductance data were recorded by BioTrace+ running on Windows (XP/Vista). The recordings were acquired without interruption during the experiment, and the data display was screened from the participants. The sampling rate was adjusted to 256 Hz.

The ECG was measured using two disposable pre-gelled Ag-AgCl electrodes that were placed on the participant’s wrists, as well as on the inner elbow. Electrodermal activity was recorded with two Ag-AgCl electrodes, placed on the middle and ring fingers of the non-dominant hand, which were sanitized with alcohol [105, 106].

Studies support that short recording times are reliable to obtain HRV measures. In the frequency domain, for high frequency measures (HF), 40–50 s seem to be sufficient to obtain accurate results [107, 108]. Given that the narrations varied in length, the paradigm of Ho et al. [109] was adopted; that is, we analysed the first 60 s from the onset of the narration, which is also within the recommended interval for SCR analysis of a minimum of 4 s and a maximum of 5 min [110–113]. In the photo induction paradigm, we analysed the full recording interval from the onset of the photo, which was 23 s in line with similar previous studies [114–116].

Heart rate values were extracted with Artiifact software [117], following two steps: 1) Manual detection of R-R peaks to identify missing or false detected R-R peaks. 2) Automatic artifacts correction using cubic interpolation method, which follows a nonlinear approach and provides better results than deletion or linear correction [118]. The interpolation method acts also as a low pass filter, and thus no further low/high pass filters were used [119–121]. The main measure was the percentage of the variance in heart rate that occurred in the high frequency range (0.15 to 0.40 Hz).

The EDA raw data were analyzed with Ledalab v.3.2.9 [110, 111, 122] using the Continuous Decomposition Analysis method to distinguish the phasic (driver) information from the underlying tonic sudomotor nerve activity. This method was followed to enable a distinction from a “zero” baseline, so any disruptions in the signal are represented as distinct fluctuations. These features offer the advantage of unbiased experimental manipulation and they are useful especially in cases with high phasic activity, whether induced by an experimental setup or relevant in a clinical context [110]. Raw EDA data were smoothed via convolution with a Hann window to reduce error noise and fitted to a bi-exponential Bateman function. Data were optimized by a conjugated gradient descent algorithm to reduce the error between them and the inbuilt skin conductance model [123]. The main measure was the mean amplitude of fluctuations (peaks) above baseline. We employed a typical threshold for peak detection of 0.05μS [124–126].

Based on Sturm [127] and Olney et al. [128] we calculated a composite unique score of overall physiological response for each participant [100, 101]. That is, we extracted the standardized scores (z- value) for the sympathetic (SCR) and parasympathetic (HRV) indexes, mean amplitude and HF, respectively. Then the absolute values were calculated, so greater values will represent higher physiological responses.

## Results

### Composite physiological activation score

#### Narration induction

**Fig 1:**
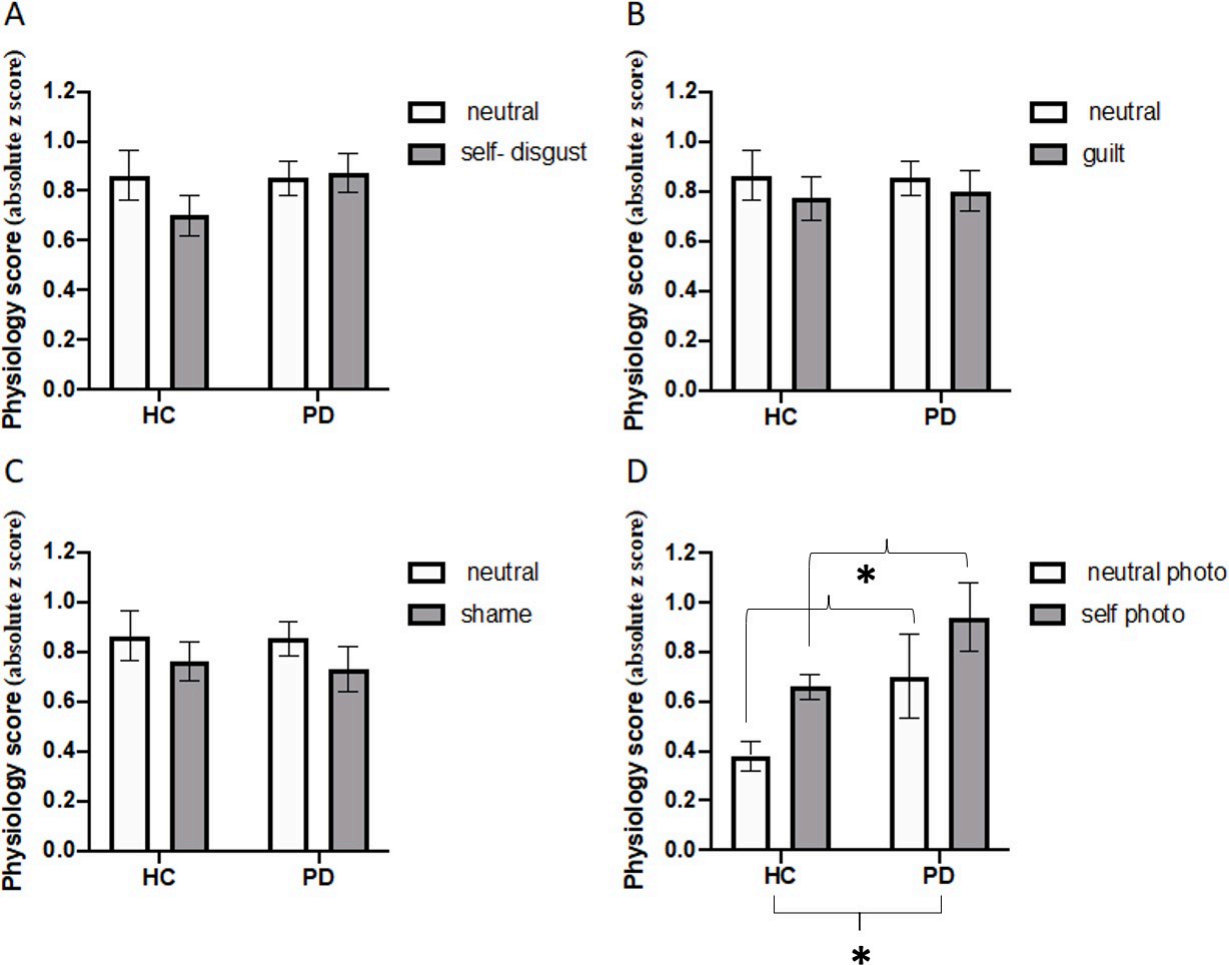
Composite physiology scores (szcores) as a function condition (emotion and neutral) and group (HC = Healthy Control, PD = Parkinson’s Disease patients). Panels A-C: Physiological response for self-disgust, guilt and shame narration paradigms, respectively. Panel D: Physiological responses for the photo paradigm (self-disgust induction). Error bars represent standard error, and asterisk significant differences (p < .05).

Composite values for self-disgust, guilt and shame were submitted to three separate 2 x 2 ANOVAs with Condition (neutral vs SCE) as the within-subjects factor and Group (HC and PD) as the between-subject factor. None of the effects reached statistical significance (see Fig 1). Self-disgust: F(1, 35) = 1.08, p = .30, ηp2 = .03, F(1, 35) = .39, p = .53, ηp2 = .01, and F(1, 35) = 2.90, p = .09, ηp2 = .07, for the main effects of Condition, Group and their interaction, respectively; Guilt: F(1, 40) = .64, p = .42, ηp2 = .01, F(1, 40) = .02, p = .87, ηp2 = .00, and F(1, 40) = .16, p = .69, ηp2 = .00, for the main effects of condition and group and their interaction, respectively; Shame: F(1, 29) = .15, p = .70, ηp2 = .00, F(1, 29) = .12, p = .72, ηp2 = .00, and F(1, 29) = .15, p = .69, ηp2 = .00, for the main effects of Condition and Group and their interaction, respectively.

#### Self-photo induction

The composite values were submitted to a 2 x 2 ANOVA with Condition (neutral vs self-photo) as the within-subjects factor and Group (HC and PD) as the between-subject factor. The main effects of Condition and Group were significant, F(1, 34) = 6.21, p = .01, ηp2 = .15, and F(1, 34) = 6.8, p = .01, ηp2 = .16, respectively (see Fig 1). That is, there was a significantly higher overall physiological response for the self-photo condition (0.79) than the neutral photo condition (0.53), and for PD patients (0.81) than for HC participants (0.52). However, the interaction between Condition and Group was not significant, F(1, 34) = .22, p = .63, ηp2 = .00. The main effect of Condition for was no longer significant when adjusting for the influence of anxiety and depressive symptoms, F(1, 33) = 3.04, p = .090, ηp2 = .035.

### SCR-mean amplitude analyses

#### Narration induction

**Fig 2:**
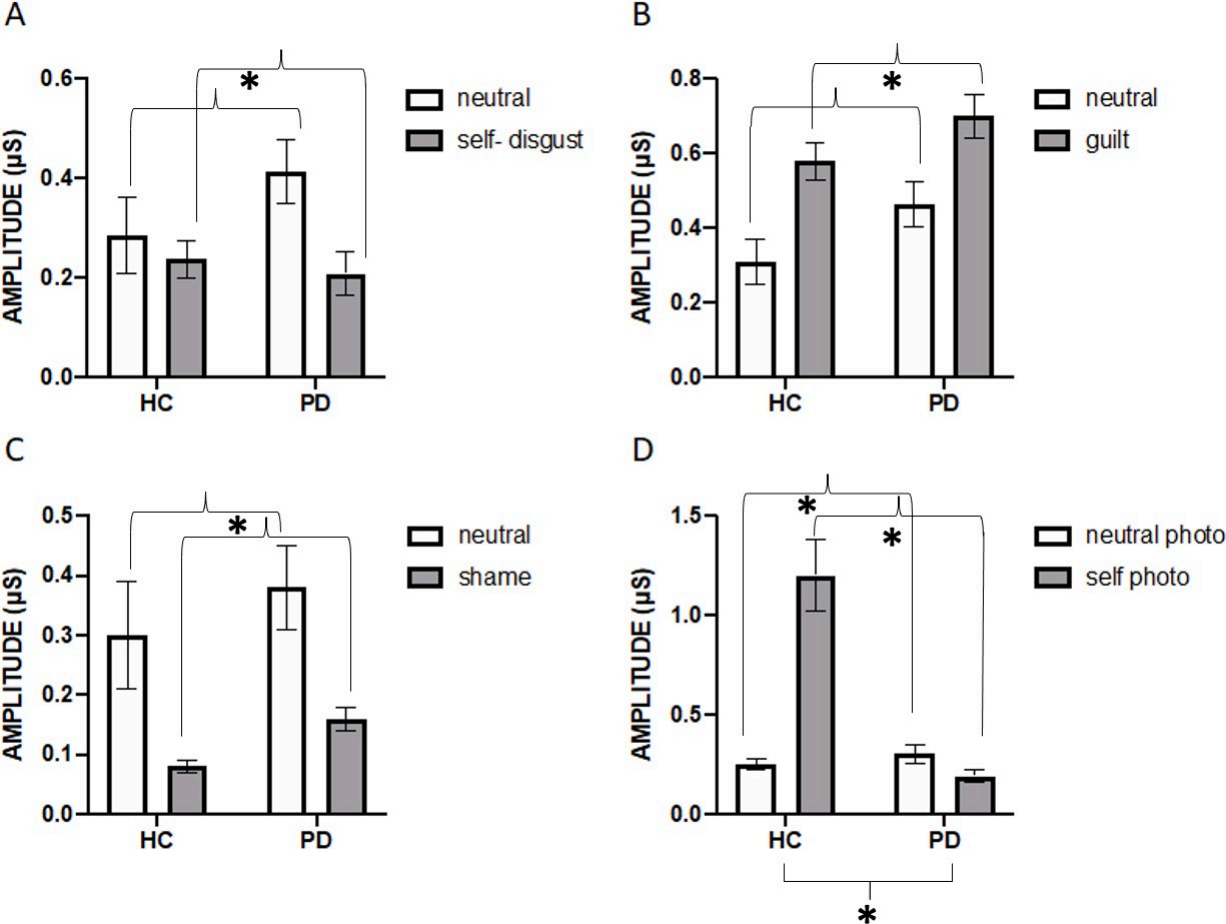
Peak amplitude (μS) of the skin conductance response as a function of condition (emotion and neutral) and group (HC = Healthy Controls and PD = Parkinson’s Disease patients). Panels A-C: Physiological responses for self-disgust, guilt and shame narration paradigms, respectively. Panel D: Physiological responses for the photo paradigm (self-disgust induction). Error bars represent standard error and asterisk significant differences (p < .05).

Mean amplitude data for self-disgust, guilt and shame were submitted to three 2 x 2 ANOVAs with Condition (neutral vs SCE) as the within-subject factor and Group (HC and PD) as the between-subject factor (see Fig 2). The main effect of Condition was significant for the three analyses, for self-disgust F(1,37) = 4.84, p = .034, ηp2 = .116, for guilt F(1,43) = 16.22, p< 0.001, ηp2 = .274, and for shame F(1,31) = 12.79, p = .001, ηp2 = .292. That is, the mean amplitude was significantly lower for the self-disgust condition (.22 μS) and the shame condition (.12 μS) than their respective neutral conditions (.35 and .34 μS, respectively, see Fig 2); whereas the mean amplitude was higher for the guilt condition (.64 μS) than for the neutral condition (.38 μS). The main effect of Condition for self-disgust was no longer significant when adjusting for the influence of anxiety and depressive symptoms, F(1, 36) = 1.79, p = 0.189, ηp2 = .023.

The main effect of Group was also significant for guilt F(1,43) = 4.32, p = .04, ηp2 = .09, but not for self-disgust, F(1,37) = .70, p = .40, ηp2 = .01, or shame, F(1,31) = 1.96, p = .17, ηp2 = .06. That is, the mean amplitude was higher for PD patients (.58 μS) than for HC participants (.44 μS). Finally, the interaction between Condition and Group was not significant for any of the analyses, F(1,37) = 1.85, p = .18, ηp2 = .04, F(1,43) = .07, p = .78, ηp2 = .00, F(1,31) = .00, p = .96, ηp2< .00, for self-disgust, guilt and shame, respectively.

#### Photo-induction paradigm

Mean amplitude data were submitted to a 2 x 2 ANOVA with Condition (neutral vs self-photo) as the within-subjects factor and Group (HC and PD) as the between-subject factor. Results showed significant main effects of Condition, F(1,56) = 15.76, p< 0.01, ηp2 = .22, and Group, F(1,56) = 17.95, p< 0.01, ηp2 = .24. That is, the mean amplitude was higher for the HC group (.76 μS) than the PD group (.24 μS), and for the self-photo condition (.73 μS) than the neutral condition (.27 μS). The interaction Condition by Group was also significant, F(1,56) = 24.13, p< 0.01, ηp2 = .30. The analysis of the interaction showed that the two groups significantly differed in the self-photo condition (MeanHC = 1.27 μS, MeanPD = .19 μS, t = -4.73 (56), p< .001), but not in the neutral condition (MeanHC = .25 μS, MeanPD = .30 μS, t = -.79 (56), p = .43). In the self-photo condition, the group of HC had larger amplitude than the PD group (see Fig 2). The main effect of Condition was no longer significant when adjusting for the influence of anxiety and depressive symptoms, F(1, 55) < .01, p = 0.995, ηp2< .001.

### HRV-HF band analyses

#### Narration induction

**Fig 3:**
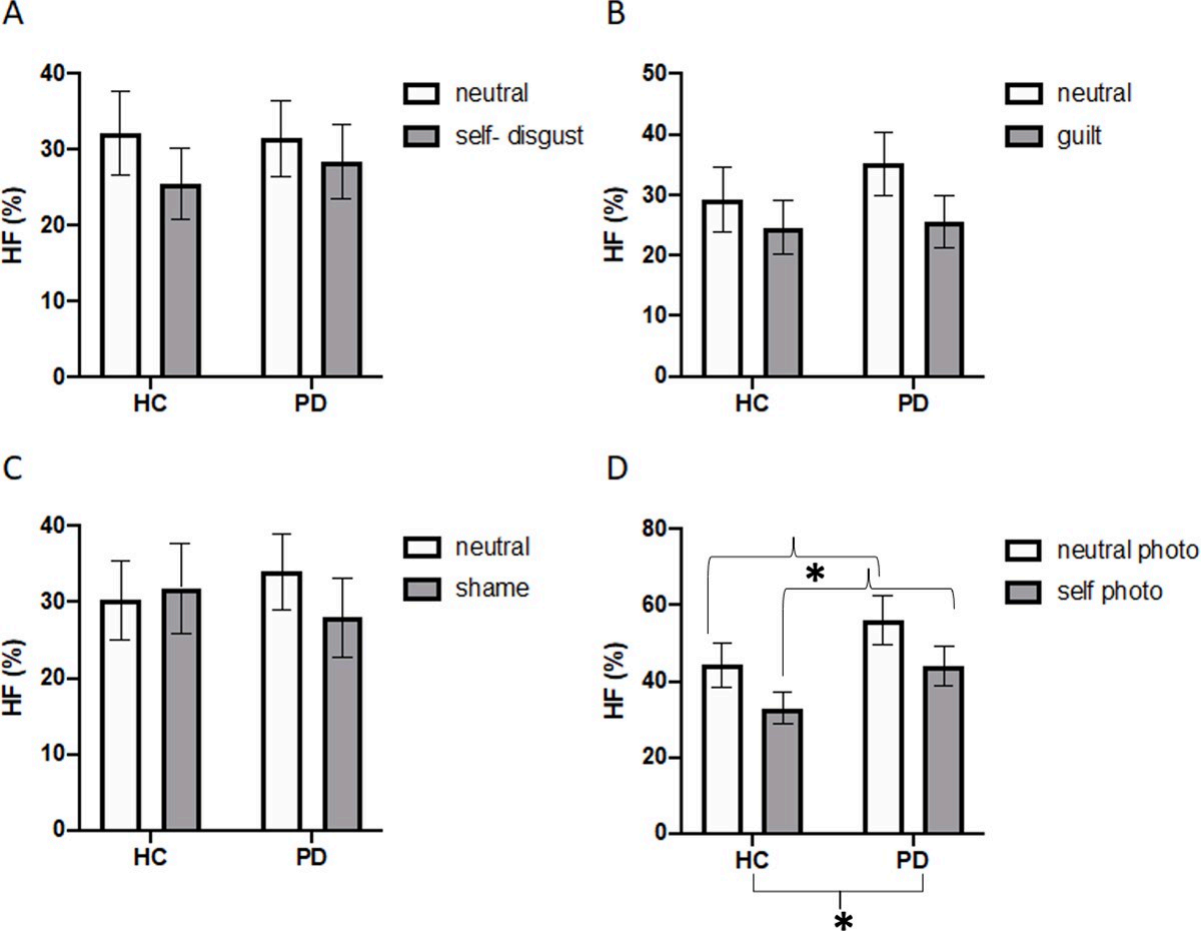
Percentage of high frequency band heart rate variability (HF) as a function of condition (emotion and neutral) and group (HC = Healthy Controls and PD = Parkinson’s Disease patients). Panels A-C: Physiological responses for self-disgust, guilt and shame narration paradigms, respectively. Panel D: Physiological responses for the photo paradigm (self-disgust induction). Error bars represent standard error and asterisk significant differences (p < .05).

Mean percentage of HF band activity for self-disgust, guilt and shame were submitted to three separate 2 x 2 ANOVAs with Condition (neutral vs SCE) as the within-subjects factor and Group (HC and PD) as the between-subject factor. None of the effects reached statistical significance (see Fig 3). Self-disgust: F(1, 43) = 1.78, p = .18, ηp2 = .04, F(1, 43) = .03, p = .85, ηp2 = .00, and F(1, 43) = .03, p = .85, ηp2< .001, for the main effects of Condition, Group and their interaction respectively. Guilt: F(1, 42) = 2.49, p = .12, ηp2 = .05, F(1, 42) = .42, p = .51, ηp2 = .01, and F(1, 42) = .30, p = .58, ηp2 = .00, for the main effects of Condition and Group and their interaction, respectively. Shame: F(1, 43) = .43, p = .51, ηp2 = .01, F(1, 43) <1, p = .99, ηp2< .001, and F(1, 43) = 1.26, p = .26, ηp2 = .02, for the main effects of Condition and Group and their interaction, respectively.

#### Photo-induction paradigm

Mean percentages οf HF band activity were submitted to a 2 x 2 ANOVA (see Fig 3). The main effects of Condition, F(1, 37) = 4.75, p = .03, ηp2 = .11, and Group, F (1, 37) = 4.28, p = .04, ηp2 = .10, were significant. That is, the percentage of HF band activity was significantly higher for PD patients (50.10%) than for HC participants (38.60%), and for the neutral photo condition (50.20%) than for the self-photo condition (38.47%). However, the interaction Condition by Group was not significant, F(1, 34) = .57, p = .45, ηp2 = .01. The main effect of Group was no longer significant when adjusting for the influence of anxiety and depressive symptoms, F(1, 37) = 1.72, p = 0.198, ηp2 = .026.

## Discussion

To determine the extent to which bottom up, biophysiological, processes may have contributed to the increased self-disgust levels in patients with Parkinson’s disease reported in Tsatali et al. [75], we examined heart rate (HRV) and skin conductance (EDA) responses [79–82] in two emotion induction paradigms; narration and photo induction. The former, based on Dickerson et al. [129], required participants to narrate past experiences of self-disgust, guilt and shame, whilst the latter was designed to elicit disgust towards self- image Overton et al. [58] by presenting self-photos [130]. Tsatali et al. [75] reported increased levels of self-reported and experimentally induced self-disgust in patients with Parkinson’s disease relative to control participants, but no differences in guilt and shame. The differences in self-disgust were significant after adjusting for the effects of depression.

Overall, the results suggest that altered bottom up biophysiological activation, in response to emotion-induction, is unlikely to have contributed to increased self-disgust in patients with Parkinson’s disease. Specifically, we found that the images in the photo induction paradigm resulted in significant changes in physiological responses in patients with Parkinson’s disease relative to healthy controls. The images produced higher composite responses, higher percentages of HRV in the high frequency range but lower SCR amplitudes. The differences in SCR for the photo induction were also significantly modulated by condition (self vs neutral image). That is, patients with Parkinson’s disease mainly differed from healthy controls in the self condition, where responses in the patient group were significantly lower. Hence, contrary to our prediction, the analysis of the interaction showed that patients had a significantly smaller sympathetic response (SCR amplitude) to the self-disgust induction than the healthy controls (see Fig 2). This suggests that the physiological response to self-disgust, as elicited by the self-photo, was actually diminished in the group of patients. The remaining differences in physiological response as a function of emotion induction condition were not modulated by group. Indeed, several of the differences in physiological response as a function of emotion induction condition were lost when adjusting for the influence of anxiety and depressive symptoms. Thus, findings do not support the hypothesis that increased self-disgust in Parkinson’s patients, relative to healthy controls, may have resulted from heightened physiological response to this emotion.

With regard to the other two emotions investigated, shame and guilt, there were significant changes in physiological responses in patients with Parkinson’s disease relative to the healthy controls, only for SCR in the guilt condition. Guilt yielded significantly higher SCR amplitudes in patients than healthy controls, whereas shame showed no differences. In line with our predictions, and the lack of significant differences between the groups in self-report measures guilt, this difference was not modulated by condition (guilt vs neutral narration). Previous research indeed indicates that elevated levels of guilt are positively correlated with augmented SCR variables [94–96]. However, our findings contrast with Harley et al. [98] who also reported elevated levels of SCR during shame. This difference might be attributed to the difference in the experimental induction paradigms, as Harley et al. [98] used questionnaires and not instructed narrations. Overall, our findings suggest that SCR may be differentially affected by guilt and shame/self-disgust. This dissociation may reflect the distinction between SCEs that are likely to trigger adaptive behaviours (e.g. apologising) to undo the harm that has been done, as with the case of guilt, or maladaptive ones, as with shame and self-disgust which are more pathogenic emotions without a clear adaptive function [39, 131, 132].

In the majority of the analyses in which the factor group did not significantly interact with the emotion-induction condition, there were nevertheless significant differences between the overall physiological response of patients with Parkinson’s disease and healthy controls. As secondary findings our results suggest that, in the main Parkinson patients had higher overall autonomic activity than matched healthy controls. The findings were consistent across the analyses; Parkinson patients had higher composite scores, higher percentages of HRV in the high frequency range in the photo-induction paradigm and SCR amplitudes in the guilt narration paradigm than control participants. These findings do not seem to be in agreement with previous studies that show decreased HRV in Parkinson’s disease due to dysautonomia, levodopa medication and progression of motor symptoms [133–136]. Some studies have also reported diminished SCR amplitude in Parkinson patients [3, 137], but others have not found differences between Parkinson patients and healthy controls in skin conductance measures [138]. Our group of patients was in early-moderate stages of Parkinson, so some of the discrepancies with previous studies may be related to progression of the disease, medication or other clinical characteristics such as depressive and anxiety symptoms.

Limitations of the study have been largely covered by Tsatali et al. [75], relating to the use of self-report measures to determine the levels of SCEs in the patients and the study’s cross sectional design. In terms of limitations that are specifically relevant to the current study however, the high level of exclusions from the participant pool due to poorer quality recordings is the principal concern. Although the cause of those cases where recording quality was poorer is unknown, electrodermal responses tend to be smaller overall in the elderly [139], and sweat gland activity differs in the elderly [140], which may lead to high epidermal resistance. Presumably, the latter can also affect heart rate recordings as well. Importantly for the present study, drop outs occurred in similar proportions from both the patient and control groups.

To conclude, the present study suggests that altered physiological response in patients with Parkinson’s disease is unlikely to contribute to the increased, experimentally induced, self-reported levels of self-disgust. Considering to the two-factor theory of emotion [76], the absence of altered physiological responses by inference suggests that the source of elevated self-disgust levels in Parkinson’s disease may instead lie at the cognitive level. Parkinson’s disease is characterised by frontal lobe degeneration [141, 142], specifically in the οrbitofrontal cortex, which plays a key role in cognitive control and emotional regulation [22–24]. Also, given dopamine’s presence in frontal areas such as the prefrontal cortex and the anterior cingulate insula [143], its absence in Parkinson’s disease is likely to affect frontal processing. Hence, not surprisingly, frontal lobe deficits have been collectively reported in Parkinson’s disease [123]. We hypothesise that because SCEs have a cognitive component [35–37], frontal lobe deficits in Parkinson’s disease may give rise to the increased levels of self-disgust in patients with the disorder. This contention is supported by previous research in neurological population (with fronto-temporal dementia and Alzheimer’s disease), which suggested that executive function may contribute to emotion regulation in the context of SCEs [144, 145]. The selective impact of Parkinson’s disease on some SCEs rather than others may relate to the specific circuitry underlying these emotions and/or the precise nature of the cognitive deficits induced by the disorder.
